# BPIFB1 (LPLUNC1) is upregulated in cystic fibrosis lung disease

**DOI:** 10.1007/s00418-012-0990-8

**Published:** 2012-07-06

**Authors:** Lynne Bingle, Kirsty Wilson, Maslinda Musa, Bianca Araujo, Doris Rassl, William A. Wallace, Elizabeth E. LeClair, Thais Mauad, Zhe Zhou, Marcus A. Mall, Colin D. Bingle

**Affiliations:** 1 Academic Unit of Oral and Maxillofacial Pathology, School of Clinical Dentistry, University of Sheffield, Sheffield, UK; 2 Academic Unit of Respiratory Medicine, Department of Infection and Immunity, University of Sheffield, Sheffield, S10 2JF UK; 3 Department of Biomolecular Sciences, Faculty of Applied Sciences, University Technology MARA, Shah Alam, Selangor Malaysia; 4 Department of Pathology, Sao Paulo University Medical School, Sao Paulo, Brazil; 5 Department of Pathology, Papworth Hospital, Cambridge, UK; 6 Department of Pathology, University of Edinburgh, Edinburgh, UK; 7 Department of Biological Sciences, DePaul University, Chicago, IL USA; 8 Division of Pediatric Pulmonology and Allergy and Cystic Fibrosis Center, Department of Pediatrics III, University of Heidelberg, Heidelberg, Germany; 9 Department of Translational Pulmonology, Translational Lung Research Center, University of Heidelberg, Heidelberg, Germany

**Keywords:** BPIFB1, LPLUNC1, Airway, Immunohistochemistry, Cystic fibrosis, Mouse models

## Abstract

Although the biology the PLUNC (recently renamed BPI fold, BPIF) family of secreted proteins is poorly understood, multiple array based studies have suggested that some are differentially expressed in lung diseases. We have examined the expression of BPIFB1 (LPLUNC1), the prototypic two-domain containing family member, in lungs from CF patients and in mouse models of CF lung disease. BPIFB1 was localized in CF lung samples along with BPIFA1, MUC5AC, CD68 and NE and directly compared to histologically normal lung tissues and that of bacterial pneumonia. We generated novel antibodies to mouse BPIF proteins to conduct similar studies on ENaC transgenic (ENaC-Tg) mice, a model for CF-like lung disease. Small airways in CF demonstrated marked epithelial staining of BPIFB1 in goblet cells but staining was absent from alveolar regions. BPIFA1 and BPIFB1 were not co-localised in the diseased lungs. In ENaC-Tg mice there was strong staining of both proteins in the airways and luminal contents. This was most marked for BPIFB1 and was noted within 2 weeks of birth. The two proteins were present in distinct cells within epithelium. BPIFB1 was readily detected in BAL from ENaC-Tg mice but was absent from wild-type mice. Alterations in the expression of BPIF proteins is associated with CF lung disease in humans and mice. It is unclear if this elevation of protein production, which results from phenotypic alteration of the cells within the diseased epithelium, plays a role in the pathogenesis of the disease.

**Electronic supplementary material:**

The online version of this article (doi:10.1007/s00418-012-0990-8) contains supplementary material, which is available to authorized users.

## Introduction

Palate lung nasal epithelium clone (PLUNC) was first described in the nasal epithelium of the mouse embryo and trachea/bronchi of adult mice (Weston et al. 1999). After cloning the human and mouse PLUNC genes (Bingle and Bingle 2000; LeClair et al. 2001), we made the key observation that PLUNC belongs to a group of proteins that make up a branch of a lipid transfer protein family (Bingle and Craven 2002, 2004; Bingle et al. 2004). Due to the complexity of this family, and conflicting gene nomenclature, a new nomenclature has been developed. Within this framework all family members have been renamed using the root symbol BPIF# for “BPI fold containing”. Family members that contain a single structural domain have the designation BPIFA (so that SPLUNC1/PLUNC becomes BPIFA1) and those containing two domains have the designation BPIFB (so that LPLUNC1 becomes BPIFB1) (Bingle et al. 2011a, b). This nomenclature has been adopted throughout this paper.

The interspecies diversity and rapid evolution of BPIF genes (Bingle et al. 2004, 2011a, b) support a role for the proteins in host defence, although functional data are only just starting to emerge (Gally et al. 2011; Lukinskiene et al. 2011; Shin et al. 2011; Wright et al. 2010). BPIFA1 has been shown to have a surfactant-like function (Gakhar et al. 2010) and also to be involved in the regulation of the amiloride-sensitive epithelial sodium channel, ENaC (Garcia-Caballero et al. 2009), an important pathway for airway surface liquid homeostasis critical for normal mucociliary clearance and thus an important mechanical innate defense mechanism of the lung (Mall 2008). BPIF proteins are predominantly localised where innate defence is a major requirement, namely in the nasal, tracheal and bronchial passages as well as in major salivary glands and minor mucosal glands of the oral cavity (Bingle and Bingle 2011). All BPIF proteins contain signal peptides and multiple proteomic studies have shown that members of the family are present in fluids from these regions (Barnes et al. 2008).

BPIF proteins have been implicated in the pathophysiology of chronic lung diseases and in particular in cystic fibrosis (CF). For example, proteomic analysis of nasal epithelial cells from CF patients has demonstrated increased levels of BPIFA1 (Roxo-Rosa et al. 2006) and BPIFA1 and BPIFB1 are increased in sputum from patients with CF (McCray et al. 2005) and epithelial cells from CF patients express abundant *BPIFA1* and *BPIFB1* (Scheetz et al. 2004). Recent observations also have implications for a biological role for the proteins in the disease. As mentioned previously, BPIFA1, but not BPIFB1, has been shown to inhibit the biochemical activation of ENaC sodium channels by preventing proteolytic processing and therefore activation of the channel (Garcia-Caballero et al. 2009; Rollins et al. 2010). As ENaC-mediated Na+/fluid absorption is increased in CF airways and because this abnormality contributes to airway surface liquid depletion, an important mechanism in the pathogenesis of CF lung disease (Mall 2009), it has been hypothesized that upregulation of BPIFA1 may counteract the basic defect and improve impaired airway surface hydration in CF (Garcia-Caballero et al. 2009). BPIFA1 is greatly increased in the small airways and plugged lumens in CF (Bingle et al. 2007). Elevated levels of BPIF proteins in CF, in the face of chronic infection and inflammation, suggest that the protein is rendered non-functional by the abnormal milieu present in the airways of CF patients. Alternatively, the protein may be upregulated as part of the innate immune defense system in the chronically infected CF lung.

Building on our previous studies of BPIFA1 and BPIFB1 in normal lung (Bingle et al. 2005, 2010) and of BPIFA1 in CF tissue (Bingle et al. 2007), the present study investigates the expression of BPIFB1 and BPIFA1 in lungs from CF patients and in bENaC-Tg mice with CF-like lung disease (Mall et al. 2004).

## Materials and methods

### Immunohistochemistry

Sections from the major bronchi and peripheral lung were cut from 15 cases of normal lung and from 10 patients with CF who were undergoing lung transplantation. Histologically disease-free control samples were from tissue resections taken during surgery for cancer, where no tumour tissue was seen to be present. The CF tissue was obtained from explanted lungs. We also studied lungs from post mortem samples from five patients who had died from bacterial pneumonia. Tissue used in this study was obtained with full ethical approval from the Department of Pathology at the University of Edinburgh, UK; Papworth Hospital, Cambridge, UK; and Sao Paulo University Hospital, Sao Paulo, Brazil and used in Sheffield with local ethical committee approval.

The slides were stained as previously described (Bingle et al. 2005, 2007, 2010). The following antibodies were used in this study: a rabbit polyclonal antibody against human BPIFA1 (Campos et al. 2004) (final dilution 1:300); rabbit polyclonal antibodies against BPIFB1 (final dilution of 1:100 for BPIFB1A and 1:600 for BPIFB1B), (Bingle et al. 2010), a polyclonal antibody to human mucin 5AC (MUC5AC, a gift from David Thornton, University of Manchester, UK; final dilution 1:250); monoclonal antibodies to CD68 and neutrophil elastase purchased from Dako (1:400 and 1:300, respectively), and a rabbit antibody against mouse CCSP (a gift from Barry Stripp, final dilution 1:1,000). Due to the low level of sequence similarity that exists between mouse and human BPIFA1 (72 %) and BPIFB1 (59 %) we developed mouse-specific antibodies to both proteins described below. Both the BPIFA1 and BPIFB1 antibodies were used at a final dilution of 1:750. A standard antigen retrieval procedure using tri-sodium citrate in a microwave for 8 min was used for the human and mouse BPIFB1 antibodies as well as the MUC5AC, CD68 and CCSP antibodies. Sections were incubated with 100 % normal serum (goat for polyclonal antibodies, horse for monoclonal antibodies) at room temperature for 30 min and then at 4 °C overnight with the antibodies diluted as indicated above with 100 % normal serum. Rabbit or mouse IgGs (DAKO) were used as negative controls on replicate slides. A Vectastain Elite ABC kit (Vector Laboratories) containing an appropriate biotin-labelled secondary antibody was used according to the manufacturer’s instructions. Peroxidase enzymatic development was performed using a Vector NovaRed substrate kit resulting in red staining in positive cells. Sections were counterstained with haematoxylin, dehydrated to xylene and mounted in DPX. Alcian blue staining of acidic mucins was performed using a standard histological method.

### Experimental animals

We used C57BL6 and C57BL6/129sv mice for examination of the normal distribution of the proteins in mice. Animals were housed in the DePaul University and the University of Sheffield animal care facilities and were allowed access to food and water ad libitum. All studies were performed with the approval of local animal care committees. We collected tissues from mice aged 6–16 weeks with some tissues being collected following perfusion fixation and all samples being fixed in cold 4 % paraformaldehyde. Fixed tissues were embedded in paraffin according to standard protocols.

The generation of βENaC-Tg mice (line 6608) has been previously described (Mall et al. 2004, 2008). The colony was maintained on a mixed genetic background (C3H/HeN × C57BL/6N), and βENaC-Tg mice were identified by PCR as described (Mall et al. 2004, 2008). To ensure there were no strain-related differences, wild-type (WT) littermates served as controls in all experiments. Mice were housed in a specific pathogen-free animal facility at the University of Heidelberg, Germany and had free access to chow and water. For BAL collection, mice were deeply anesthetized via intra-peritoneal injection of a combination of ketamine/xylazine (120 and 16 mg/kg, respectively), the trachea cannulated, and lungs lavaged with PBS. Samples were centrifuged and the cell-free BAL fluid was stored at −80 °C. For immunohistochemical analysis, lungs that had not been subjected to lavage were removed from mice, through a median sternotomy. Lungs were fixed in 10 % formalin buffered saline, paraffin embedded and sectioned. Six WT and six βENaC-Tg mice at each of two time points (2 and 6 weeks of age) were used to generate the samples. All experimental animal studies were approved by the Regierungspräsidium Karlsruhe, Germany.

### Western blotting

5 μl aliquots of mouse BAL from the βENaC-Tg mice and WT littermate controls, were denatured, resolved on 12 % SDS-PAGE gels and western blotted using specific antibodies against mouse BPIFA1 and BPIFB1 (1:500 dilution). Detection was performed using ECL and X-ray film (Amersham) following incubation with a HRP conjugated secondary antibody (1:2,000 dilution).

Human peripheral blood neutrophils and mononuclear (MNC) cells from normal healthy donors were isolated by density gradient centrifugation and cultured as previously described (Savill et al. 1989). Monocyte-derived macrophages (MDM) were differentiated from monocytes on tissue culture plastic using standard protocols (Dockrell et al. 2001). Protein samples from a given number of purified human neutrophils and MDM were resolved on 12 % SDS-PAGE gels. 5 μl of apical secretions from primary human tracheobronchial epithelial cells differentiated at an air liquid interface (ALI) was used as a positive control (Bingle et al. 2007). Blots were probed with antibodies to human BPIFA1 and BPIFB1 (1:500) and subsequently re-probed with a polyclonal antibody to human Mcl-1 (sc-819, Santa Cruz Biotechnology Inc), a Bcl-2 family member expressed in myeloid cells (Bingle et al. 2000). This was to confirm the presence of protein in the neutrophil and MDM lanes. Detection was performed as above.

## Results

### BPIFB1 is increased in CF airways

**Fig. 1:**
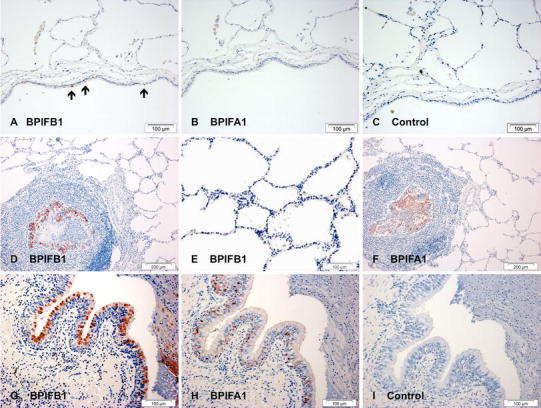
Increased BPIFB1 and BPIFA1 in CF lung. Immunohistochemistry for BPIFB1 (**a**, **d**, **e**, **g**) and BPIFA1 (**b**, **f**, **h**) was performed as described in "Materials and methods". Sections show staining in samples of normal large airway (**a**–**c**) and in sections from patients with cystic fibrosis (**d**–**i**). Sections **c** and **i** are controls (no primary antibody). The *black arrows* in panel **a** indicated BPIFB1 positive cells in the normal airway. *Scale bars* are present on each individual panel

We confirmed our previous results which showed that expression of BPIFB1 was limited to a small number of airway cells in histologically normal sections of large bronchi (shown by black arrows in Fig. 1a) but was absent from peripheral lung and alveolar macrophages. Expression of BPIFA1 was essentially absent from the same regions (Fig. 1b). The use of no antibody control slides helped to confirm the specificity of the staining (Fig. 1c). In cases of CF, BPIFB1 staining was present in the epithelium lining the distended and inflamed airways (Fig. 1d, g). Again no staining was seen in the peripheral lung tissue (Fig. 1d, e). Although both proteins are localized to similar regions, i.e. conducting airways but not alveoli (Fig. 1f), they stain different cells (Fig. 1g, h). The BPIFB1 positive cells have the characteristics of goblet cells whereas BPIFA1 positive cells are more basally distributed and probably represent non-ciliated cells. In keeping with the fact that BPIF proteins are secreted, both proteins were often localised to the luminal contents (Fig. 1d, f), which contain both inflammatory cells and mucoid secretions. The specificity of BPIFB1 staining in the CF cases was confirmed using a second affinity-purified antibody (BPIFB1A) (Bingle et al. 2010) (Supplementary figure 1).

**Fig. 2:**
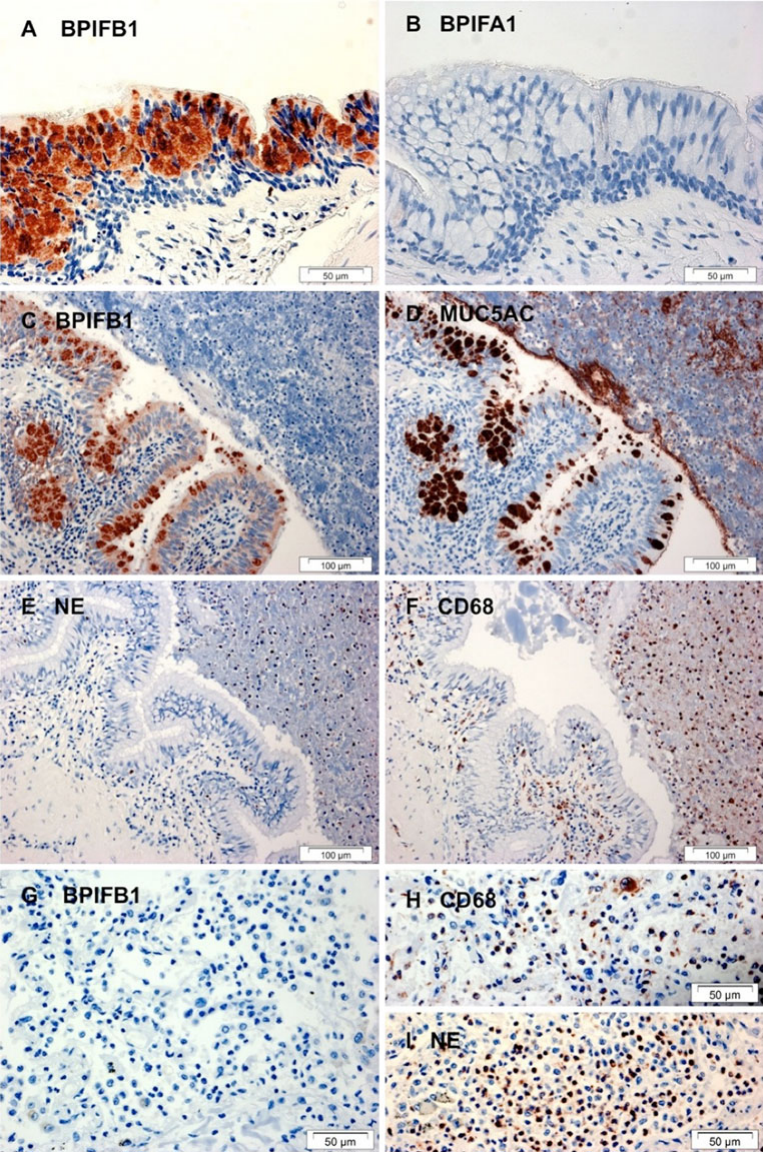
BPIFB1 is predominantly expressed in a goblet cell population in CF airways and is absent from neutrophils and macrophages. Immunohistochemistry for BPIFB1 (**a**, **c**, **g**), BPIFA1 (**b**), MUC5AC (**d**) NE (**e**, **i**) and CD68 (**f**, **h**) was performed as described in "Materials and methods" using sections from patients with cystic fibrosis (**a**–**f**) or from bacterial pneumonia (**g**–**i**). *Scale bars* are present on each individual panel

Goblet cell expression of BPIFB1 was confirmed (Fig. 2a, c) by co-localisation with the goblet cell marker protein MUC5AC (Fig. 2d), whereas these cells were negative for BPIFA1 (Fig. 2b). Sections were stained for neutrophil elastase to identify neutrophils and CD68 for macrophages, (Fig. 2e, f), and this confirmed that inflammatory cells did not express BPIFB1 in the CF lung. Macrophages and neutrophils in lungs from bacterial pneumonia also did not express BPIFB1 (Fig. 2g–i). BPIFB1 and BPIFA1 were also undetectable by western blotting of human neutrophil and macrophage lysates (Supplementary figure 2).

As patients with CF have significant airway remodelling associated with repeated chronic bacterial infections, it is unclear what is responsible for the elevated production of BPIFB1 in the disease. The severity of the lung disease in the patient samples available also makes it difficult to study differential expression of the proteins in the early stage of disease. To address this issue, we studied the localisation of BPIF proteins in the lungs of mice with a CF-like lung disease.

### BPIFB1 and BPIFA1 are differentially expressed in the respiratory tract of mice

**Fig. 3:**
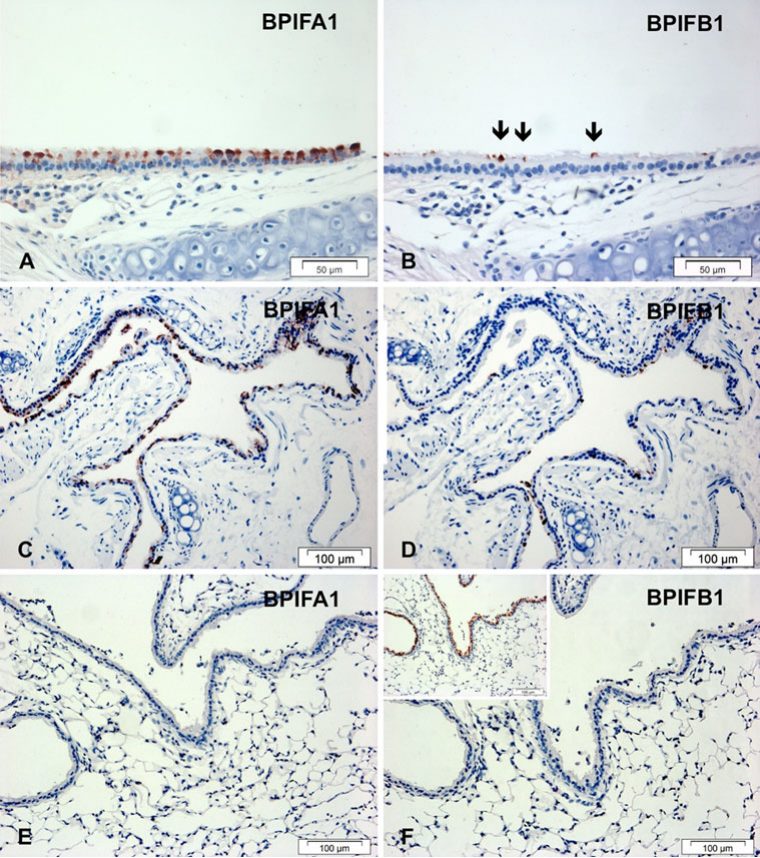
BPIFA1 and BPIFB1 do not co-localise in the adult mouse respiratory tract. Immunohistochemistry was performed as described in "Materials and methods" using antibodies specific for murine BPIFA1 (**a**, **c**, **e**) and BPIFB1 (**b**, **d**, **f**). Sections show serial samples of the trachea (**a**, **b**), large airways (**c**, **d**) and smaller airways and peripheral lung (**e**, **f**). The inset figure in **f** represents staining of a further section with the Clara cell marker, SCGB1A1 (CCSP). The *black arrows* identify the rare BPIFB1 positive cells in the normal mouse trachea. *Scale bars* are present on each individual panel

We initially localised BPIFB1 and BPIFA1 in lung sections from WT mice using novel species-specific antibodies. BPIFA1 was strongly expressed in the non-ciliated epithelial cells of the trachea (Fig. 3a, and Supplementary figure 3C, D) but was not co-localised with acid mucins as shown by distinct Alcian blue staining in the mucous cells of the tracheal submucosal glands (SMG) (Supplementary figure 3A, B). BPIFB1 staining was seen in a much smaller population of non-ciliated epithelial cells than BPIFA1 (shown by black arrows in Fig. 3b). These cells may represent the rare goblet cells seen in normal mouse trachea. Airway SMGs were negative for BPIFB1 (not shown). In more distal sections of the airways, BPIFA1 staining was more prominent than that of BPIFB1 (Fig. 3c, d). As airways became smaller, and within the peripheral lung, staining of both proteins was lost (Fig. 3e, f), whereas the Clara cell marker, SCGB1A1, was seen throughout the smaller airways (Fig. 3f, inset).

### Expression of BPIFB1 and BPIFA1 is increased in a mouse model of CF lung disease

Having established that mouse BPIF proteins had similar distribution patterns to that seen in humans, we determined their expression in a well-established murine CF model, the ENaC-Tg mouse which phenocopies the airway surface liquid depletion and impaired mucus clearance seen in CF (Mall et al. 2004; Zhou et al. 2011).

**Fig. 4:**
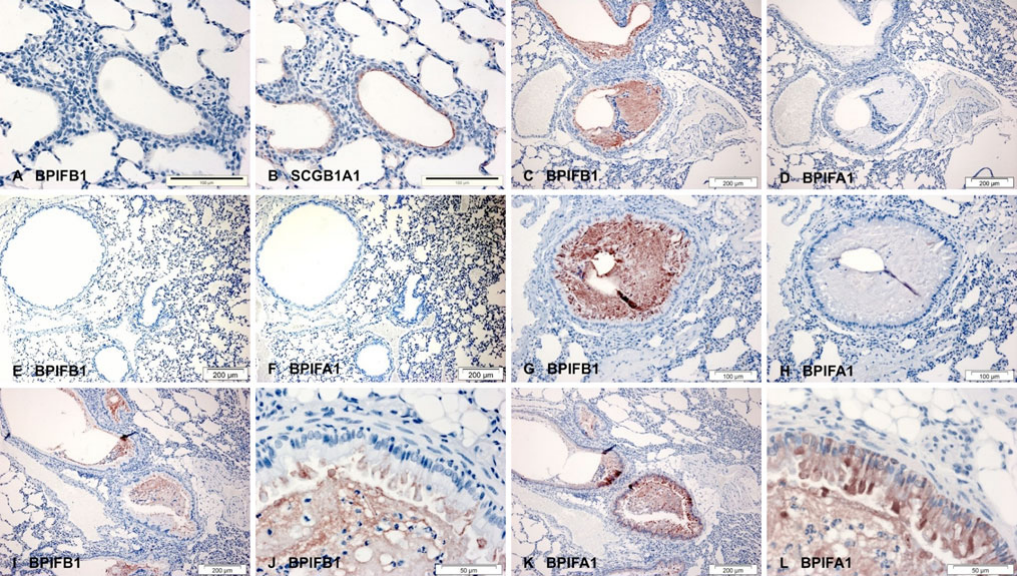
BPIFB1 and BPIFA1 are increased in the occluded airways of *scgb1a1*-ENaC transgenic mice. Immunohistochemistry was performed as described in "Materials and methods" using antibodies specific for murine BPIFB1 (**a**, **c**, **e**, **g**, **i**, **j**) and BPIFA1 (**d**, **f**, **h**, **k**, **l**) and SCGB1A1 (**b**). Sections show serial samples of lungs from 2 week old wild type mice (**a**, **b**), 2 week old transgenic mice (**c**, **d**) 6 week old wild type mice (**e**–**f**) and 6 week old transgenic mice (**g**–**l**). *Scale bars* are present on each individual panel

Lungs from 2-week-old WT mice were negative for BPIFB1 (Fig. 4a) whereas SCGB1A1 (which drives expression of the transgene) stained the surface airway epithelium (Fig. 4b). Neither BPIFB1 nor BPIFA1 were seen in 6-week-old WT lungs (Fig. 4e, f). In marked contrast there was significant BPIFB1 staining in lungs from 2- and 6-week-old ENaC-Tg mice (Fig. 4c, g, i, j) with the protein being seen in both the epithelial cells lining the airways and in the luminal contents. Strong staining of BPIFA1 was also seen in some of the ENaC-Tg lungs (compare Fig. 4k, l with d, h). Close examination of the sections revealed that BPIFA1 stained a greater number of cells than did BPIFB1 (Fig. 4j, l) but it is not clear from our data if the proteins co-localise.

**Fig. 5:**
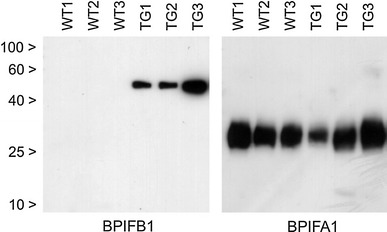
BPIFB1 is significantly increased in the BAL fluid of *Scgb1a1*-ENaC transgenic mice. Replicate SDS-PAGE gels of BAL fluid samples from three wild type and three transgenic mice (both at 6 weeks of age) were generated as outlined in "Materials and methods". The resultant blots were subjected to western blotting and detected with specific antibodies against mouse BPIFB1 and BPIFA1 as described. The position of the molecular mass markers are indicated by the *black arrows*

BAL from 6-week-old transgenic mice contains readily detectable BPIFB1 whereas samples from age-matched wild-type littermate controls do not (Fig. 5). In contrast, BPIFA1 was present in similar amounts in both transgenic and wild-type BAL fluid samples. Importantly, neither protein exhibited any evidence of significant proteolysis.

## Discussion

Although the prototypic two-domain BPIF protein, BPIFB1, has been identified in multiple proteomic and genomic studies (Barnes et al. 2008), information on the tissue localisation of the protein and its role in lung biology and disease is very limited (Bingle et al. 2010). As BPIF proteins have been increasingly implicated in the pathogenesis of CF (Garcia-Caballero et al. 2009; Roxo-Rosa et al. 2006; McCray et al. 2005; Bingle et al. 2007) we undertook to study BPIFB1 in both diseased human tissue and in an established mouse model of CF.

Strikingly, BPIFB1 is greatly increased in the chronically inflamed lungs of patients with CF, a condition that is characterised by mucous cell hyperplasia (Burgel et al. 2007; Henke et al. 2007). Coupled with repeated cycles of chronic infection, CF lung disease results in thickened mucoid secretions that contain multiple inflammatory cells and harbour a host of pathogens. Our data show that in CF, BPIFB1 is produced in goblet cells in regions of the lung that co-express MUC5AC and the protein is part of the viscous complex that occludes the airways. BPIFB1 (and BPIFA1) has previously been shown to be associated with the mucus-containing fraction of BAL lavage fluid and sputum (Kesimer et al. 2009) and it seems likely that it makes up part of the protective shield that overlies the airway epithelium. Although BPIFA1 has been implicated in regulating ENaC activity and airway surface liquid to counteract the CF defect (Garcia-Caballero et al. 2009; Rollins et al. 2010), the function of BPIFB1 in this location remains unknown.

Despite the significant levels of BPIFB1 staining seen in the airway epithelium in CF, there was no staining in the peripheral lung. We have previously reported increased BPIFA1 protein in airway epithelial cells in CF (Bingle et al. 2007) but, similar to the situation seen with BPIFB1, it was not detected in the peripheral lung. Importantly, BPIFB1 is not co-localised with BPIFA1 in CF. This replicates the situation we have described in non-diseased lung tissue (Bingle et al. 2005, 2010) and shows that the mechanisms which govern the spatial regulation of these two genes is retained in CF. Although the mechanisms that govern the expression of BPIF proteins have not been elucidated, we and others have shown that both proteins are significantly expressed in tracheobronchial epithelial cells differentiated at the ALI and that their expression is modulated by the differentiation status of the cultures (Bingle et al. 2007, 2010; Ross et al. 2007). We have not shown that BPIFB1 is a direct transcriptional target of pro-inflammatory mediators, including IL-1β, TNFα and bacterial LPS (data not shown). A proteomic study (Candiano et al. 2007) also showed no significant increase of BPIFB1 following cytokine treatment of ALI cells. Our results suggest that it is the phenotypic alteration in the epithelium, potentially induced by airway surface liquid depletion in CF airways that leads to the increase in both proteins and is not, therefore, the response of a single epithelial cell type in the diseased lung.

BPIFB1 is not produced by inflammatory cells present in the luminal contents of the diseased lung, or by those resident within the lung tissues. Due to the nature of the lung tissue in CF, it is difficult to differentiate between staining of BPIF proteins in the mucus surrounding the inflammatory cells or within the cells themselves. In addition BPIFA1 has also been reported to be present in neutrophil granules (Bartlett et al. 2008). We addressed these issues using cases of bacterial pneumonia where significant numbers of neutrophils and macrophages were seen but where no BPIFB1 staining was noted. We also failed to detect BPIFB1 and BPIFA1 in MDMs and peripheral blood neutrophils by western blotting. These data, along with lack of identification of either protein in multiple neutrophil and macrophage proteomic datasets (Bewley et al. 2011; Kraft-Terry and Gendelman 2011; Lietzén et al. 2011), strongly support our view that BPIF proteins are not produced at significant levels by cells of the myeloid lineage, but that epithelial cells secrete both proteins into the lumen.

This paper is the first to show localisation of either BPIFA1 or BPIFB1 in the mouse lung. *Bpifa1* was originally identified in developing nasal passages and the upper respiratory tract of adult mice (Weston et al. 1999). Our data confirm that the upper airway is a major site of protein production. The staining appears to be in a non-ciliated cell population, even though the protein is clearly associated with the ciliated cell surface in both locations. Expression of BPIFA1 does not always co-localise with the Clara cell marker SCGB1A1 (CCSP) in the airway, as expression of BPIFA1 is lost in the more distal Clara cells, which retain very strong expression of SCGB1A1. Further evidence that the protein is produced in non-ciliated cells comes from the observation that strong staining of BPIFA1 is still seen in mice lacking ciliated cells due to the deletion of foxj1 (data not shown). This cellular localisation is consistent with that seen for the human proteins (Bingle et al. 2005, 2010).

BPIFB1 is present in what appears to be goblet cells within the large, conducting airways. Staining in the trachea and upper airways for BPIFB1 is therefore more limited than for BPIFA1 as there are few goblet cells present in the airways of healthy mice (Livraghi et al. 2009). Goblet cells in other locations, including for example the GI tract and the conjunctiva, do not stain for BPIFB1 (data not shown). One of the important findings from our study is that in the normal mouse lung expression of BPIFB1 is very limited. This is consistent with the very limited reports of BPIFB1 being found in proteomic datasets of mouse BAL (Guo et al. 2005; Gharib et al. 2010) as well as our observations that limited detectable protein is present in BAL from normal mice (Fig. 5). The lack of staining of either protein in the normal mouse lung parenchyma also mirrors the situation in humans (Bingle et al. 2005, 2010).

The data from the lungs of βENaC-Tg mice suggest that it is the airway surface lining depletion-induced airway disease and epithelial remodeling, rather than the loss of CFTR function and/or chronic infection per se, that is responsible for the differential expression of BPIFB1 in the CF lung. As expected from previous studies (Wilke et al. 2011), *Cftr* ^*tm1Unc*^ mice showed no evidence of CF-like lung disease, and perhaps not unexpectedly, analysis of lung tissues revealed that there were no readily detectable differences in the staining intensity or localization of BPIF proteins (data not shown). In contrast to this, and again mirroring the data seen in human CF, we found significant BPIFB1 staining associated with CF-like mucus obstruction, goblet cell metaplasia and inflammation in the airways of the βENaC-Tg mice. In this model, overexpression of βENaC in the airway epithelium causes increased airway Na+ absorption that results in airway surface liquid depletion. This in turn causes a reduction in mucus transport due to increased viscosity and to the development of a spontaneous CF-like lung disease with the characteristic airway mucus obstruction and chronic airway inflammation (Mall et al. 2004, 2008; Zhou et al. 2011). Unlike the situation seen in human CF, this model is not associated with chronic bacterial infections and, therefore, should prove to be useful in further understanding the function of BPIF proteins in the pathogenesis of CF lung disease. At this time it is unclear why the induced expression of BPIFA1 in this model is not seen in all βENaC-Tg mice, but our assumption is that animals that express more BPIFA1 are likely to have a lesser number of clara cells than those with lower expression. This requires more quantitative analysis.

In summary, we have shown a significant increase in BPIFB1 in the airways of patients with CF as well as in a murine model of the disease. It is unclear if the increased production of BPIF proteins in CF influences the development of lung disease but it seems likely that the increased production of these putative host defence proteins will be a response to the epithelial remodeling that accompanies this condition. The nature of our studies does not allow us to determine if increased BPIF production is associated with disease severity but as the proteins are readily detectable in BAL fluid, the development of quantitative assays will enable its role as a potential biomarker for disease severity to be studied.

## Electronic supplementary material

Below is the link to the electronic supplementary material.

**Supplementary Fig 1 Specificity of staining with BPIFB1 antibodies.** Immunohistochemistry was performed on serial sections of a CF cases as described in materials and methods section using two specific for BPIFB1 antibodies generated against distinct epitopes in the protein. A. BPIFB1 abB (used for all of the other Figures) and B, BPIFB1 abA. (B). (JPEG 620 kb)

**Sup Figure 2 Lack of expression of BPIFB1 and BPIFA1 in neutrophils and monocyte derived macrophages (MDMs).** Protein samples equivalent to 1x106 or 2.5x106 neutrophils, 1x106 MDMs (either mock treated or infected with Neisseria meningitidis mc58) as well as a positive control sample of ALI secretion (2l) were resolved on replicate 12% SDS-PAGE gels and western blotted using polyclonal antibodies against BPIFB1, BPIFA1 and the myeloid enriched Bcl 2 family member, Mcl-1. The position of the molecular mass markers are indicated by the black arrows. (JPEG 88 kb)

**Supplementary Fig 3 BPIFA1 is localised to non-ciliated epithelial cells in the upper respiratory tract.** Immunohistochemistry was performed on mouse sections as described in materials and methods section using antibodies specific for murine BPIFA1. Sections show samples of adult trachea (A, B, D,) and nasal septal epithelium (C). Alcian Blue staing showed that BPIFA1 was not present in mucous cells of the submucosal glands (A, B). Scale bars are present on each individual panel. (JPEG 584 kb)
