# Supplementary figures and images for: BPIFB1 (LPLUNC1) is upregulated in cystic fibrosis lung disease
Source: Histochem Cell Biol. 2012 Jul 6;138(5):749–58. doi: 10.1007/s00418-012-0990-8 (PMC3470695; doi:10.1007/s00418-012-0990-8)

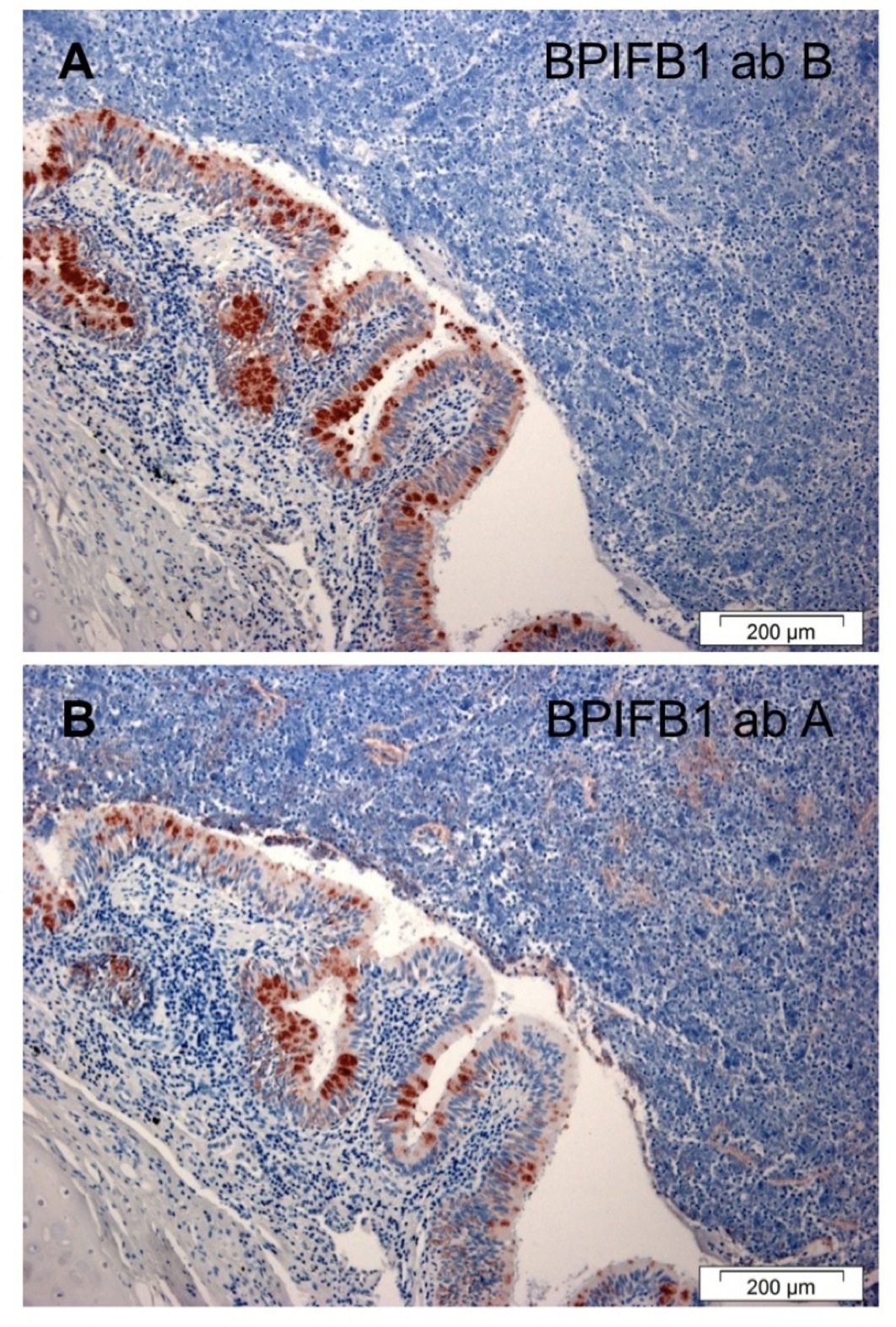

Supplement: Supplementary file 1 — Supplementary Fig 1 Specificity of staining with BPIFB1 antibodies. Immunohistochemistry was performed on serial sections of a CF cases as described in materials and methods section using two specific for BPIFB1 antibodies generated against distinct epitopes in the protein. A. BPIFB1 abB (used for all of the other Figures) and B, BPIFB1 abA. (B). (JPEG 620 kb) [file 418_2012_990_MOESM1_ESM.jpg]

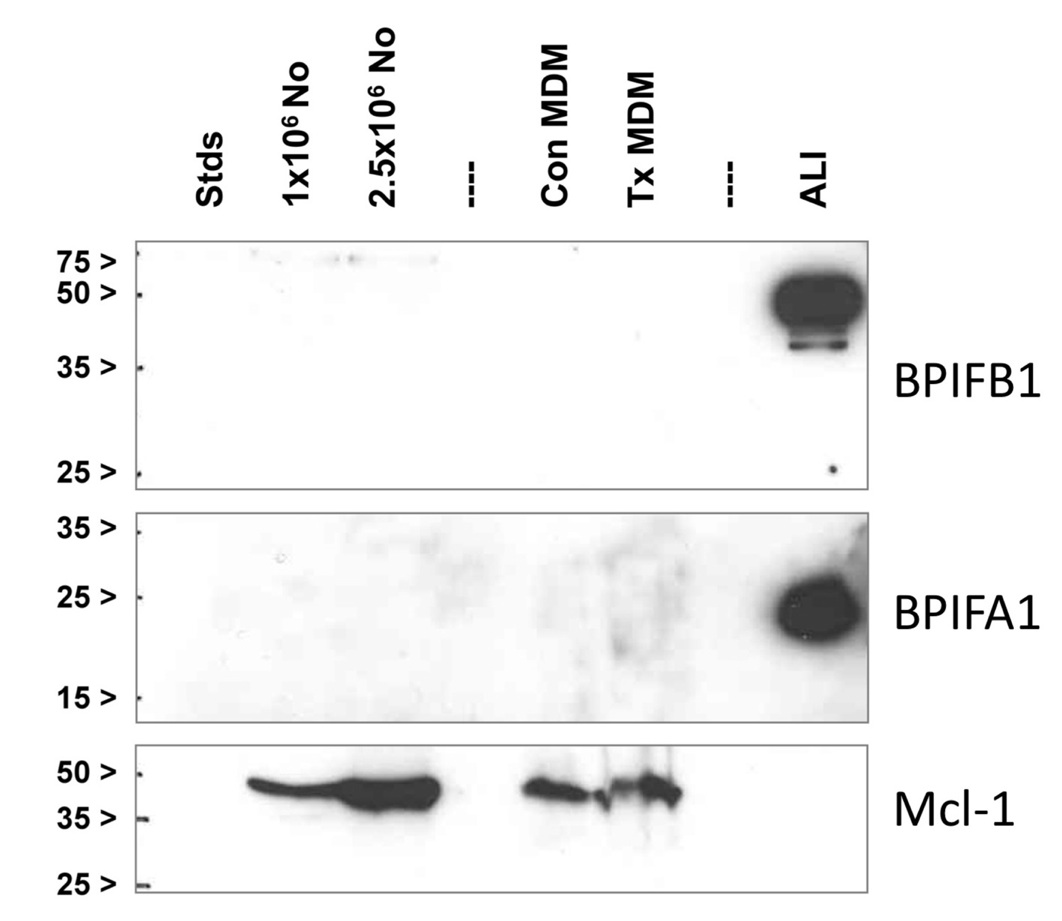

Supplement: Supplementary file 2 — Sup Figure 2 Lack of expression of BPIFB1 and BPIFA1 in neutrophils and monocyte derived macrophages (MDMs). Protein samples equivalent to 1x10^6^ or 2.5x10^6^ neutrophils, 1x10^6^ MDMs (either mock treated or infected with Neisseria meningitidis mc58) as well as a positive control sample of ALI secretion (2l) were resolved on replicate 12% SDS-PAGE gels and western blotted using polyclonal antibodies against BPIFB1, BPIFA1 and the myeloid enriched Bcl 2 family member, Mcl-1. The position of the molecular mass markers are indicated by the black arrows. (JPEG 88 kb) [file 418_2012_990_MOESM2_ESM.jpg]

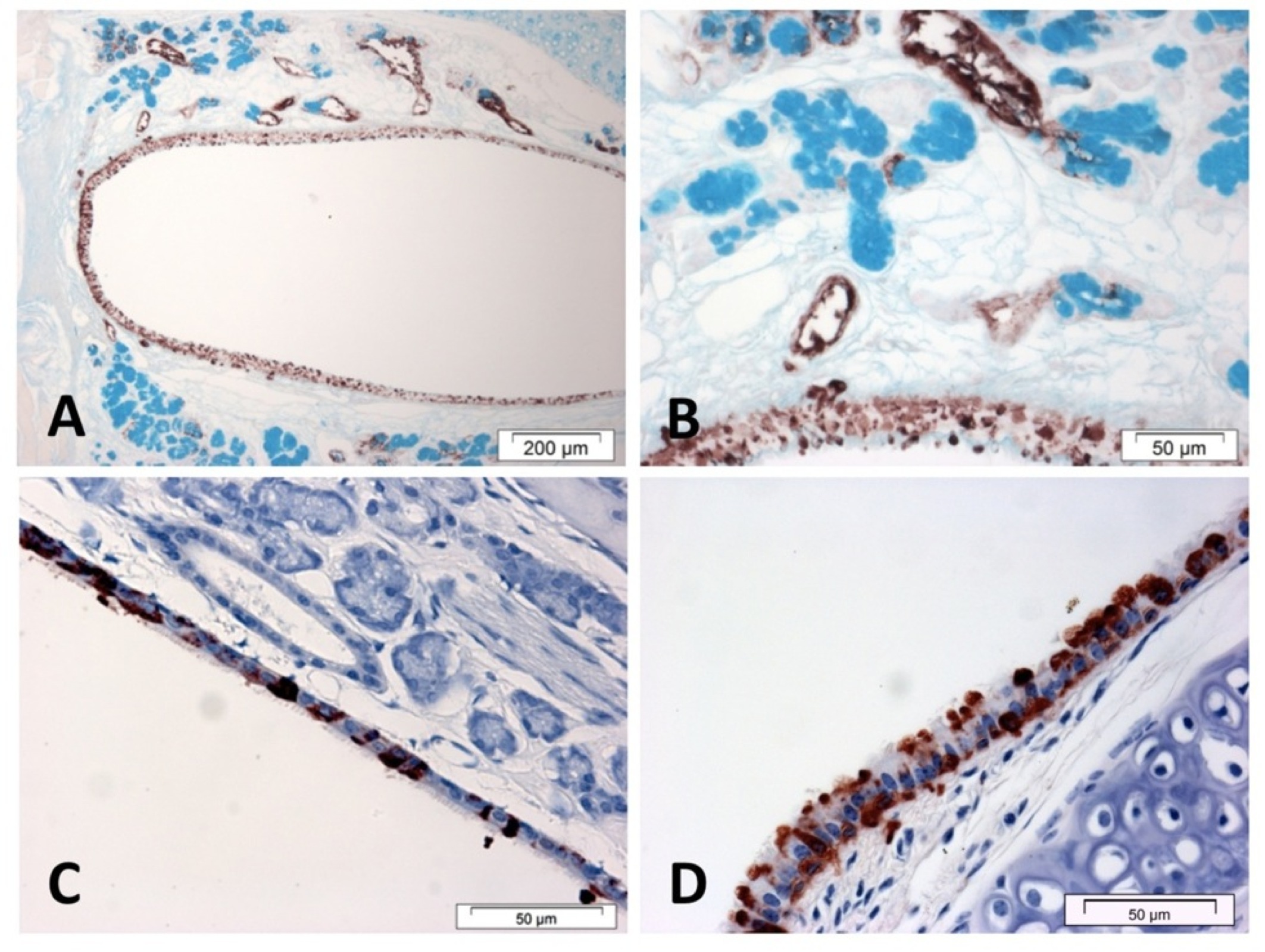

Supplement: Supplementary file 3 — Supplementary Fig 3 BPIFA1 is localised to non-ciliated epithelial cells in the upper respiratory tract. Immunohistochemistry was performed on mouse sections as described in materials and methods section using antibodies specific for murine BPIFA1. Sections show samples of adult trachea (A, B, D,) and nasal septal epithelium (C). Alcian Blue staing showed that BPIFA1 was not present in mucous cells of the submucosal glands (A, B). Scale bars are present on each individual panel. (JPEG 584 kb) [file 418_2012_990_MOESM3_ESM.jpg]
